# PW02-038 - Treatment-resistant NOMID with autoantibodies

**DOI:** 10.1186/1546-0096-11-S1-A179

**Published:** 2013-11-08

**Authors:** L Broderick, J Chang, I Szer, HM Hoffman

**Affiliations:** 1 Medicine, University of California San Diego, La Jolla, USA; 2 Rady Children's Hospital San Diego, San Diego, USA; 3 Pediatrics, University of California San Diego, La Jolla, USA; 4 Ludwig Institute of Cancer Research, San Diego, USA

## Introduction

Neonatal Onset Multisystem Inflammatory Disorder (NOMID) is the most severe of the Cryopyrin-Associated Periodic Syndromes (CAPS) spectrum [1]. Given the persistent nature of CAPS, symptoms may be confused with autoimmune diseases. However, unlike autoimmune diseases, autoinflammatory disorders are not associated with high-titer autoantibodies or antigen-specific T cells. Here we describe a patient with severe and early onset features of NOMID confirmed by NLRP3 mutation, hepatitis, neonatal antibodies and a limited response to high dose anakinra.

## Case report

N.D. is a 2.5 yo male with congenital hearing loss who initially presented at 2 months of age with direct hyperbilirubinemia and transaminitis. Liver biopsy showed giant cell hepatitis. He had a positive ANA (with strong cytoplasmic staining), anti-smooth muscle antibody, and mildly positive double-stranded DNA antibody although his mother had no autoimmune antibodies. At 3 months of age, he developed persistent fevers and an urticarial-like rash and at 1 year he developed hip arthritis followed by knee swelling. His physical exam was notable for poor growth, macrocephaly, and irritability. He had generalized lymphadenopathy, pronounced hepatosplenomegaly and arthralgia with effusion of the left knee. Laboratory evaluations demonstrated persistently elevated CRP up to 200 mg/L, ESR 76 mm/hr, and ferritin 114 ng/mL. Urine showed no proteinuria or mevalonic acid. MRI of the brain demonstrated severe volume loss and Xray of distal femur showed physeal irregularity. Skin biopsy showed neutrophilic urticaria particularly surrounding eccrine glands. Sequencing of NLRP3 revealed a c.926T>A mutation (Phe209Leu) which was described in a NOMID patient [1]. Despite treatment with anakinra, his clinical course has been complicated by uveitis, anemia, rib fractures with rachitic changes suggestive of vitamin D-deficiency rickets, chronic diarrhea and fat malabsorption, abnormal hearing, left knee reactive synovitis, seizures and significant developmental delay in speech, and both fine and gross motor skills. While he demonstrated an initial modest improvement on 4mg/kg anakinra [2,3] including decreased rash, improved mobility and development, and slow but steady weight gain, he continues to have intermittent rashes, irritability and enlarging head circumference despite dose escalation to 11mg/kg. In addition, he has a severe, persistent unilateral knee synovitis with minimal response to therapy.

## Discussion

This complex patient with evidence for persistent inflammation emphasizes the variable clinical presentation of the CAPS spectrum. The severity of this case also illustrates that NLRP3-related inflammation may begin *in utero* and suggests that in some cases, autoinflammation and autoimmunity may not be mutually exclusive.

## Competing interests

L. Broderick: None declared, J. Chang: None declared, I. Szer: None declared, H. Hoffman Consultant for: Sobi, Novartis and Regeneron
